# Agri-food wastes as substrates for oyster mushroom (*Pleurotus ostreatus*) cultivation and their agricultural potential

**DOI:** 10.1038/s41598-025-26843-y

**Published:** 2025-11-28

**Authors:** Katarzyna Miśkiewicz, Dorota Gendaszewska, Katarzyna Sieczyńska, Beata Gutarowska

**Affiliations:** 1 Łukasiewicz Research Network-Lodz Institute of Technology, 19/27 Marii Sklodowskiej - Curie Street, Lodz, 90-570 Poland; 2 Interdisciplinary Doctoral School, Lodz University of Technology, 116 Stefana Zeromskiego Street, Lodz, 90-543 Poland; 3 Faculty of Biotechnology and Food Sciences, Lodz University of Technology, 171/173 Wolczanska Street, Lodz, 90-530 Poland

**Keywords:** *Pleurotus ostreatus*, Spent mushroom substrate, Agricultural waste, Biostimulation potential, Agri-food waste decomposition, Fungal biology, Fungal physiology

## Abstract

**Fungi of the** ***Pleurotus*** **genus**,** are known for their health-promoting properties. They are grown on a wide range of lignocellulosic substrates**,** including i.e. straw**,** rice hulls and corn residues. There are numerous literature reports proving that the composition of the culture substrate affects the growth rate and yield of oyster mushrooms. The aim of the present study was to optimize the mixture of the culture medium**,** composed of agro-industrial waste**,** for the cultivation of** ***Pleurotus ostreatus*** **in order to achieve maximum yield and the fastest mycelial growth. In addition**,** the chemical composition of selected wastes was determined and the possibility of using spent** ***P. ostreatus*** **substrate as a biostimulant for plant growth was assessed. The best results in terms of yield**,** mycelial growth rate**,** and time required for substrate colonization**,** primordia formation**,** and first harvest**,** were obtained for straw with spent brewery grains and for straw with wheat bran in a proportion of 70/30%**,** while the mixture of wheat straw**,** wheat bran and sugar beet pulp in a proportion of 50/25/25% gave slightly poorer results. The maximum yield of 51.7 g/bag and the fastest growth was obtained for the substrate with the addition of spent brewery grains. Substrate formula 70% wheat straw**,** with 30% addition of spent brewery grains**,** gave the fastest time for substrate colonization**,** primordia formation and the first harvest (16 days**,** 20 days**,** 28 days respectively). The study carried out by ICP-Spectrometry technique confirmed that the** ***Pleurotus*** **spent substrate is a source of valuable elements (Ca**,** Cu**,** Fe**,** K**,** P**,** Mg**,** Mn**,** S)**,** indicating its biostimulatory potential in agriculture. Enzyme activity associated with substrate (carbohydrate) degradation**,** performed using a commercial API ZYM assay increased significantly in straw with spent brewery grains and straw with wheat bran mixture. The highest total levels of degradation products were examined by photometric method using commercially available Hach Lange cuvette tests. The highest total content of** ***Pleurotus*** **substrate degradation products**,** including organic acids**,** orthophosphate**,** ammonia nitrogen**,** nitrate**,** and nitrite**,** was found in the substrate supplemented with brewery grains. Their levels increased significantly compared to the control**,** indicating enhanced degradation activity in this substrate. Substrate after cultivation of higher fungi (Spent Mushroom Substrate-SMS) contains large amounts of valuable elements and can affect the growth and yield of crops. Their amount varies depending on the substrate used and depends on the availability of sources of C and N. Studies of the growth rate of** ***P. ostreatus*** **on a wide range of lignocellulosic substrates**,** i.e. straw**,** corn residues**,** rice husks**,** among others**,** are known. The authors of the present study extended these studies to evaluate the biodegradation of waste materials that have not yet been used in the culture of higher fungi (brewery spent grain**,** wheat bran**,** beet pulp)**,** making a significant contribution to the discipline. This study supports the implementation of the Sustainable Development Goals**,** by promoting the valorization of agro-industrial waste through sustainable biotechnological processes.**

**Supplementary Information:**

The online version contains supplementary material available at 10.1038/s41598-025-26843-y.

## Introduction

 The *Pleurotus* species are commercially important edible mushrooms grown around the world, especially in Southeast Asia, India, Europe and Africa^1,2^. They are prized, not only for their unique taste and aroma, but also for their great nutritional and medicinal value^3,4^. In Poland, one of the most appreciated mushrooms is the oyster mushroom - *Pleurotus ostreatus.* Its annual sales are estimated at approximately 3,300 tons^5^. Due to its nutritional value, ease of cultivation, and growing consumer interest in plant-based foods, its market potential continues to expand. The fruiting bodies of this mushroom are not only a good source of protein, but also carbohydrates, minerals, or vitamins^2,6–9^. Studies have shown that the mycelium of *P. ostreatus* contains high concentrations of mineral salts such as potassium, phosphorus, calcium, iron, copper, zinc, magnesium and selenium^10,11^. Moreover, the protein of this mushroom is characterized by a high content of essential amino acids e.g. leucine, lysine and phenylalanine, which are not synthesized by the human body and must be supplied in large quantities through diet^12^. Oyster mushrooms (*Pleurotus* spp., with *Pleurotus ostreatus* being the most common species) are particularly preferred for cultivation due to their shorter growth time and low requirements compared to other edible mushrooms. This leads to a higher conversion rate of substrates into fruiting bodies, increasing profitability and enabling low-cost cultivation methods^13^.

Oyster mushrooms can be successfully cultivated on various lignocellulosic substrates, such as wheat straw, soybean straw^14^, palm oil waste, bark, rice straw, corn cobs, finger millet straw, cotton waste, bamboo waste^15–17^, sugarcane bagasse^18^ or various types of wood^13^. Masevhe (2023) proved that there are about 200 types of waste from which edible mushrooms can be produced^19^. Such a wide range of raw materials that can be used to grow oyster mushrooms is due to their extensive enzymatic apparatus, which facilitates biodegradation. However, the most used substrates in the cultivation of *P. ostreatus* are straw, sawdust, and rice husk^20^. The yield ability, as well as the quality of the fruiting bodies produced, depends largely on the chemical composition and elemental content of the substrate used for cultivation^8,9^. Irregular mycelial growth resulting from improper substrate selection often results in low yields and reduced nutrient content of the mushrooms^21^. Therefore, in the mass production of *Pleurotus*, the preparation and selection of substrates represent crucial stages that often demand technical knowledge, practical experience, and adequate infrastructure; however, the use of commercially available optimized substrates offers a convenient alternative, albeit with increased financial investment. A survey of producers in Sao Paulo between 2009 and 2014 found that inadequate knowledge of substrate preparation and lack of adequate technical support posed significant challenges for oyster mushroom growers^22^.

Despite the fact that higher fungi are a valuable source of food, a large amount of waste from their production is generated worldwide. Each kilogram of mushroom produced results in 5–6 kg of by-product in the form of so-called spent mushroom substrate (SMS)^23,24^. The main strategy for disposing of SMS after cultivation has been landfilling, but this is now banned in the European Union under the Council Directive on the Landfill of Biodegradable Waste^25^. Since SMS still contains lignocellulosic biomass, it would be more economical and environmentally friendly to use it as a substrate in the processing of value-added products, e.g., as animal feed, biopreparation for soil fertility enrichment or biogas^26^. The use of SMS in agriculture mainly contributes to the improvement of soil quality, chemical adsorption of organic and inorganic pollutants, and serves as a good carrier for plant growth promoting microorganisms (PGPMs) and shows the best biological efficacy against soil and plant pathogens^27^. Therefore, integrating SMS into sustainable agricultural practices not only supports waste valorization but also can enhance ecosystem resilience and long-term soil health. To this end, it is essential to know its properties in detail, which will allow the selection of appropriate methods for its use.

The aim of the present study was to optimize the composition of the culture medium for the cultivation of *P. ostreatus* to obtain the maximum yield and the fastest mycelial growth. In addition, it was important to evaluate its biostimulatory potential for crop plants, based on the analysis of the valuable components content. Various mixtures of substrates, consisting of straw, spent brewery grains, sawdust, rapeseed meal, wheat bran and sugar beet pulp mixed in different proportions of the aforementioned, were prepared. The mixtures on which the fastest growth and yield were observed were destined for further research. The chemical composition of the selected waste compositions was determined in detail, and the possibility of using *P. ostreatus* spent substrate as a bio stimulant for plant growth was evaluated, based on the evaluation of the content of macro and micro elements, the enzymatic activity of the strain during the decomposition of selected wastes. SMS valorization is crucial for the development of sustainable mushroom cultivation within the framework of the circular economy model. Although the idea of using agricultural wastes to cultivate *Pleurotus* species is not completely new, the specific waste mixture tested (spent brewery grains, wheat bran, beet pulp) and the extensive evaluation of their effects on yield and enzyme activity bring new insights into the process itself. The research conducted in this paper provides valuable data on the biostimulatory potential of spent mushroom substrate (SMS), an area within which few literature reports exist.

## Materials and methods

### Biological material

The material for the study was a commercially available liquid culture of the oyster mushroom *Pleurotus ostreatus* (PO) purchased from a mushroom breeding company: Planto (Skrbensko, Poland).

### Granular mycelium Preparation

Oat grains obtained from organic farming near Rzeszow, Poland (49º 59′ 45.801″ N, 22º 08′ 37.093″ E) were pre-soaked in hot water (70–80 °C) for about 18 h. After this time, the grains were transferred to a sieve and allowed to drain for about 15 min. Approximately 600 g were then packed in bags with a micro filter to allow air exchange and closed hermetically. The bags were sterilized in an autoclave at 121 °C for 30 min. This procedure achieved a moisture content of the culture medium of 65% for the grains used in the study. Following the cooling phase, approximately 2 mL of the *Pleurotus ostreatus* liquid culture was aseptically introduced into the culture bag via an injection port using a sterile syringe. The grain substrate prepared in this manner was subsequently incubated in cultivation chambers at a minimum temperature of 21 °C and a relative humidity of 55 ± 5%, in complete darkness, until full colonization of the spent substrate was achieved.

### Substrates origin

The following waste substrates were used in the study: wheat straw (WS), beech wood sawdust (BS), rapeseed meal (RM), sugar beet pulp (SBP), wheat bran (WB) and brewery spent grains (BSG). Spent brewery grains came from a beer production plant in Piotrkow Trybunalski, Poland (51º 22’ 57.217” N 19º 40’ 41.313” E); wheat straw from a farm near Lodz, Poland (51º 46’ 42.671” N 18º 29’ 8.339” E), rapeseed meal from food processing plant in Rokiciny, Poland (51º 39’ 52.796” N 19º 48’ 23.761” E), beet pulp from sugar mill, Werbkowice, Poland (50º 45’ 41.208” N 23º 46’ 30.324” E) while wheat bran came from an organic farm, Tuczki, Poland (53º 21’ 8.505” N 19º 57’ 4.47” E).

### Proximate analysis of the waste substrates

The waste’s physical and chemical properties including dry matter, ash and dry organic matter contents were examined. Dry weight was determined by the dryer method (105 °C) in laboratory incubator CLW 115 (Pol-Eko, Poland). The ash content was determined by the weight method after burning in a laboratory furnance SX – muffle (Chemland, Poland). All tests were performed in triplicate.

### Substrate preparation

 The waste was dried at no less than 50 °C and then crushed. The straw was cut into 2–4 cm pieces, while the remaining waste already had a fine, loose structure (< 3 mm) and did not require additional size reduction. The dry wastes were mixed with each other in the appropriate weight/weight ratio, and straw and sawdust were used as the basis for composing the substrates for cultivation. The experiment was conducted in a completely randomized design using a factorial scheme involving 14 groups of substrate types (A-K, including subgroups), each composed of different mixtures of agro-industrial waste materials such as wheat straw, beech sawdust, sugar beet pulp, rapeseed meal, wheat bran, and brewer’s spent grains (Table 1). In total, 50 unique substrate formulations, including two controls (100% wheat straw and 100% sawdust), were tested. Each substrate variant was prepared in triplicate, resulting in 150 experimental units. A detailed description of the waste mixtures is given in Table S1 (supplementary material). After mixing, the waste was flooded with tap water and soaked for 24 h at room temperature. After this time, the finished mixes were transferred to a filter bale and drained to a moisture content of approximately 60–70%.

### Cultivation of *Pleurotus ostreatus* in test tubes

The prepared waste mixtures were transferred to test tubes (3/4 of the test tube height) and autoclaved in TINGET 45 L autoclave (Tinget, Poland) at 121 °C for 30 min. When cooled, the waste was inoculated with granular mycelium prepared earlier, at a ratio of 12 ± 2% to the wet weight of the substrate after sterilization. Tubes of inoculated medium were incubated for a period of 21 days at a temperature of not less than 21 °C. Growth rate was measured by linear examination, the thickness of the overgrown layer of the substrate (Fig. S1- supplementary materials). All tests were performed in triplicate. The control media were the straw and sawdust alone, without supplementation. The data from linear growth measurements, and the recorded time to full colonization of different substrate mixture were used to calculate the average mycelial growth rate (AGR) according to the formula 1:

1 $${\text{AGR}}\,{\text{=}}\,{\text{Linear growth of mycelium }}\left( {{\text{cm}}} \right){\text{ / Days after inoculation to total substrate colonization}}$$

### Cultivation of *Pleurotus ostreatus* in bags

The selected substrates, on which the fastest growth in tubes was observed, were prepared again. They were soaked for 24 h in tap water at room temperature, drained, and then packed in polyethylene plastic bags with a micro filter to prevent contamination from the outside. The samples were sterilized in an autoclave at 121 °C for 30 min. After cooling, the substrates were inoculated with granular mycelium and were incubated in grow boxes at no less than 21 °C, with humidity RH = 55 ± 5% in the dark, until the waste substrate was completely overgrown. After this time, the bags were placed in the incubation room at 17 ± 1 °C, RH = 75 ± 5%, with access to light during the day cycle, in order to accelerate the production of fruiting bodies. The substrate was prepared in accordance with Patent Application No. P.448,802 dated 12.06.2024^28^. The cultivation conditions were optimized as described in the previous work^29^. All tests were performed in triplicate. The time from inoculation to the first harvest was measured and recorded. Harvested fruiting bodies were weighed, stipe length, cap diameter and number of effective fruiting bodies per cluster were measured. All tests were performed in triplicate. At the end of the harvesting period, the collected data were used to calculate the total yield and biological efficiency index^30^, according to Eq. 2.

2 $$\:\text{B}\text{E}=\frac{\text{M}\text{F}\text{W}}{\text{S}\text{D}\text{W}}\text{x}100{\%}$$

where:

BE- biological efficiency,

MFW- mushroom fresh weight,

SDW- substrate dry weight.

### Determination of C, N and physicochemical characteristics of substrate and fruiting bodies

The *P. ostreatus* spent substrate and fruiting bodies from first flush were dried at 50 °C and milled in a laboratory grinder. Dry weight was determined by the dryer method. The ash content was determined by the weight method after burning in a laboratory furnance SX – muffle (Chemland, Poland). The control substrates were waste mixtures before inoculation with granular mycelium. The determination of carbon and nitrogen content of fungal samples and *Pleurotus* spent mushroom substrate was carried out using a Vario MACRO elemental analyzer CHN ELEMENTAR Analyses System GmbH, using the catalytic combustion technique in an analyzer with a TCD detector (TCD thermal conductivity detector, Sartorius M2P microbalance: weighing range 0.001–2 g, test sample weight: 1–150 mg, detection range: C: 0.02–100 mg, N 0.04–50 mg). By-product gases and measured components were separated, and detection was based on measurement of thermal conductivity of combustion products. Representative samples were analyzed in triplicate.

### ICP-OES analysis of substrate and fruiting bodies

Determination of the elemental content of Ca, Cu, Fe, Hg, K, Mg, Mn, Ni, P, Pb, S, Zn in various types of fungal culture media was carried out by the Inductively Coupled Plasma Optical Emission Spectrometry technique - ICP-OES 5110 spectrometer (Agilent, Santa Clara, USA). Samples weighing approximately 0.2 g were placed in a Teflon dish and about 7 cm^3^ of HNO_3_ (65% by weight, Chempur, Poland) was added. They were then subjected to mineralization using a Magnum II microwave mineralizer (Ertec, Wroclaw, Poland). The mineralisation process was carried out using maximum microwave power 600 W in three cycles lasting a total of 20 min, reaching a maximum temperature of 300 °C and a pressure of up to 45 bar. The resulting clear mineralisates were quantitatively transferred to 25 cm³ volumetric flasks and diluted to volume with demineralised water. A reagent blank was prepared in the same manner. The content of the test elements in the samples was read from standard curves of standards of individual metals (Ca, Cu, Fe, Hg, K, Mg, Mn, Ni, P, Pb, Zn in 5% HNO_3_ r-er, LGC Standard, Manchester, USA) and standard (S, in H2O, CPAChem, Stara Zagora, Bulgaria) by appropriate dilution of standards with 5% HNO_3_ (v/v). The concentration of the calibration solutions for ICP-OES analysis ranged from 0.005 to 50 mg·L^−1^ for the trace elements Ca (line 317,933 nm), Cu (327,395 nm), Fe (238,204 nm), Hg (184,887 nm), K (766,491 nm), Mg (279, 553 nm), Mn (257,610 nm), Ni (231,604 nm), P (213,618 nm), Pb (220,353 nm), S (181,972 nm), Zn (213,857 nm). The tests were conducted according to the test procedure. Prior to testing, the method was optimized. Analytical lines and operating mode optimal for the determination of elements were selected. Samples were analyzed by inductively coupled plasma atomic emission spectrometry using an Agilent ICP-OES 5110 spectrometer. Spectrometer parameters during analysis: generator power − 1400 W, plasma gas (flow) − 12 L·min^−1^, aux gas (flow) − 1 L·min^−1^, nebulizer gas (flow) − 0.5 L·min^−1^, nebulizer type - OneNeb, mist chamber - Double Pass Cyclonic Chamber, measurement reading time − 3 × 15 s, sample flow − 1.4 mL·min^−1^, correlation coefficient limit (0.9990). Representative samples were analyzed in triplicate. The control substrates were waste mixtures before inoculation with granular mycelium.

### Chemical analysis of extract from *P. ostreatus* post-culture substrate

*P. ostreatus* post-culture medium was poured into 50 ml of demineralized water. They were shaken on a rotary shaker ES-20 (Biosan, Poland) for 24 h at 160 rpm. The extracts were then filtered through sterile gauze and centrifuged at 6000 rpm· for 10 min in laboratory centrifuge (Ohaus, Poland). The supernatant was poured into sterile tubes. The samples were kept frozen until testing. Enzyme activity testing was performed using a commercial API ZYM assay (Biomerieux, France). The prepared extracts were spotted, in the amount of 65 µl into all wells and 1 drop each of ZYM A and ZYM B reagents. The plates were placed in a humid chamber that was included with the API ZYM kit. They were incubated at a temperature of 37 °C for 4 h. The results were read by evaluating the intensity of the color reaction on a scale from 0 to 5, where 0 is no activity and 5 is the highest activity of enzyme. Heatmaps were generated using the highest enzymatic activity values recorded among three experimental replicates. Detection of degradation products: orthophosphate, nitrate, nitrite, organic acids for the prepared extracts was performed using Hach Lange cuvette tests, according to the test manufacturer’s instructions. Photometric detection was carried out using a Hach Lange DR 6000 UV-VIS spectrophotometer (accuracy ± 1 nm, resolution ± 0.1 nm), at wavelengths specified for each determination. Representative samples were analyzed in triplicate.

### Statistical analysis of the results

The assumption of normality was checked based on the Shapiro-Wilk Test (α = 0.05). Determinations were made in triplicate, and averages were used to verify significant differences using the Tukey-Kramer test at the 0.05% significance level. Tukey’s procedure was applied to compare treatment means after ANOVA. The relationship between substrate composition and average growth rate (AGR) was examined using Principal Component Analysis (PCA) with Z-score standardization. Six substrate components (WS, BS, RM, SBP, WB, BSG) and AGR values were included in the analysis (*n* = 47 formulations). PCA loadings and sample coordinates were calculated to identify the main sources of variation and visualize substrate performance patterns relative to AGR. To assess the strength and direction of associations between substrate composition parameters (C/N ratio, nitrogen content, carbon content, organic dry matter, ash content, dry matter) and mushroom cultivation performance indicators (mushroom weight, number of fruiting bodies, first harvest, stipe length, cap diameter, total colonization, primordia formation, Biological Efficiency) a Spearman’s rank correlation analysis was conducted. A Spearman’s rank correlation analysis was conducted also to evaluate the relationships substrate physicochemical properties (C/N ratio, nitrogen content, carbon content), substrate decomposition products (organic acids, orthophosphate, ammonia nitrogen, nitrate, and nitrite) level and *P. ostreatus* enzymatic activity.Hierarchical clustering analysis: Substrate similarity was assessed using hierarchical clustering with Euclidean distance and complete linkage method. Two datasets were analyzed: (i) basic parameters including cultivation metrics and substrate composition (14 variables, *n* = 3), and (ii) extended parameters including enzymatic activities E2-E20, chemical composition, and nitrogen/phosphorus compounds (27 variables reduced to 22 after removing constant-value parameters, *n* = 3). Parameters were Z-score normalized prior to clustering to ensure equal weighting. Results were visualized as heatmaps with dendrograms showing relationships between substrate formulations and parameter groupings. Separate dendrograms were generated for both substrate samples and measured parameters to illustrate clustering hierarchy and distances.

## Results and discussion

### Physical and chemical properties of initial waste

The following waste substrates were used in the study: wheat straw, beech wood sawdust, rapeseed meal, sugar beet pulp, wheat bran and spent brewery grains as a material for oyster mushroom cultivation. These substrates were chosen due to their easy availability and excessive residues of these wastes in given production processes. The results of selected physical and chemical properties of the initial waste used in the experiment are shown in Table 1. Statistically significant differences were observed between the type of waste material and the content of dry matter, ash and dry organic matter.

**Table 1:** Physical and chemical properties of the obtained waste (± means standard deviation for *N* = 3). Values for single element designated with the same letter are not significantly different (*P* < 0.05) according to tukey’s Kramer test for multiple comparisons.

Waste substrate (Code)	Characteristic	Dry matter [%]	Ash content [%]	Organic dry matter [%]
Wheat straw (WS)	Light yellow dried stalks after wheat cultivation, without grain	92.50 ± 0.02a	2.482 ± 0.80a	90.04 ± 0.80a
Beech wood sawdust (BS)	Crushed beech wood fragments	93.26 ± 0.01a	0.53 ± 0.09b	92.74 ± 0.09b
Rapeseed meal (RM)	Bright yellow waste after rapeseed oil pressing	89.43 ± 0.01b	7.36 ± 0.02c	82.07 ± 0.01c
Sugar beet pulp (SBP)	Dark red beet processing waste	93.10 ± 0.02a	4.71 ± 0.07d	88.40 ± 0.06d
Wheat bran (WB)	Bright yellow small petals without husks	89.62 ± 0.02b	4.92 ± 0.07e	84.70 ± 0.08e
Brewery spent grain (BSG)	Brown waste from beer production	93.11 ± 0.03a	4.28 ± 0.05f	88.83 ± 0.02d

The growth of oyster mushrooms depends on the content of organic compounds; therefore, the most important parameter is the organic dry matter content. The organic dry matter content in the tested waste materials ranged from 82.07 ± 0.01% to 92.74 ± 0.09%. The highest organic dry matter content, exceeding 90%, was found in beech wood sawdust and wheat straw, which suggests that these components should remain the base of the substrate mixture for oyster mushroom cultivation. High levels of organic dry matter, above 88%, were also observed in sugar beet pulp and brewers’ spent grains. The lowest organic dry matter content was recorded in rapeseed meal, which should be supplemented with another organic substrate for effective oyster mushroom cultivation. Previous research has shown that oyster mushrooms can be successfully cultivated on various lignocellulosic substrates, such as straw, palm oil waste, bark, pomace, thresh, cotton, or wood and even olive leaves^13–17^. However, the proposed waste mixtures under consideration have not yet been tested.

### Oyster mushroom growth on waste substrate

 The linear growth kinetics of *Pleurotus ostreatus* are presented in the growth curves shown in Fig. S2 (supplementary materials). Mycelium demonstrated remarkably consistent linear patterns across all substrate compositions (A-K), with correlation coefficients (R²) exceeding 0.9 in all cases, indicating highly predictable colonization dynamics under controlled conditions. The consistently high R² values (> 0.97) across all tested formulations confirm that mycelial growth follows predictable linear kinetics during the exponential colonization phase, enabling reliable modeling of substrate utilization efficiency. The growth rates, calculated as the slope of the linear regression (Table S2, supplementary materials), ranged from 0.4026 to 0.8529 h⁻¹, with the highest observed for the J2 variant and the lowest for the K4 variant.

**Fig. 1:**
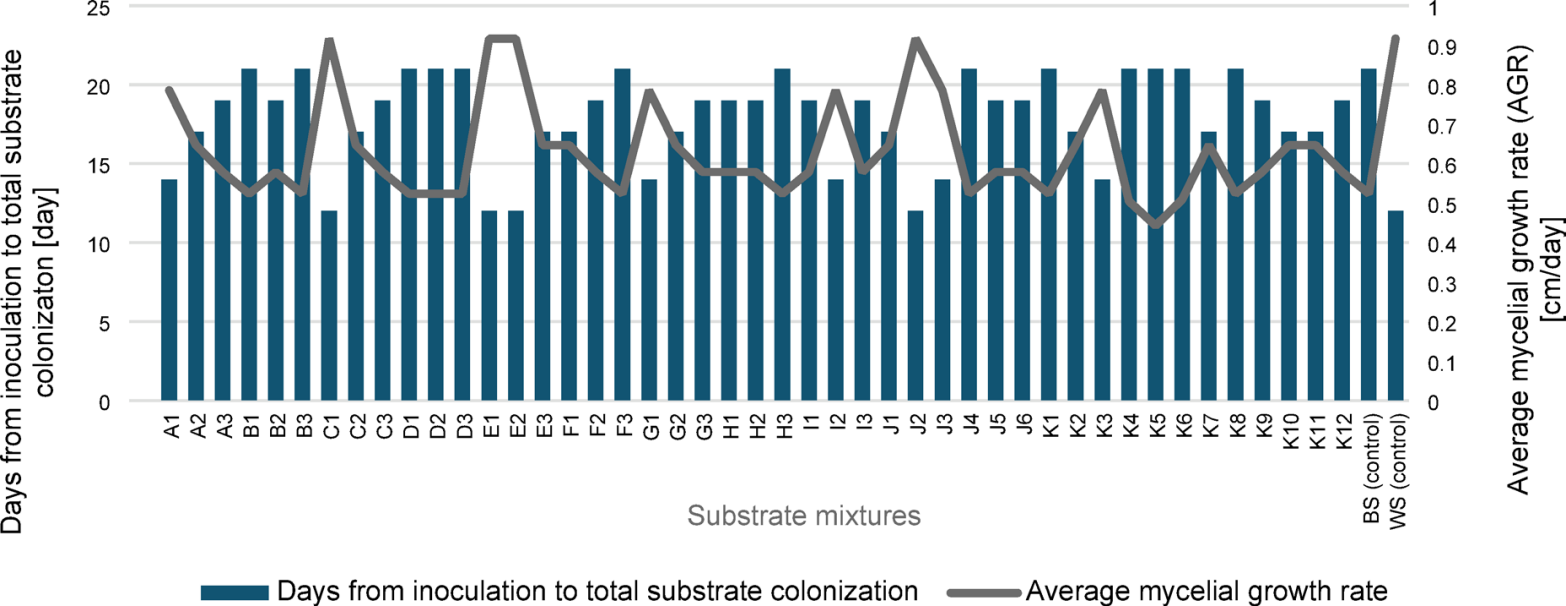
Total colonization period (in days) and average mycelial growth rate (in cm/day) of *Pleurotus ostreatus* cultivated on different substrate mixtures. Bars represent the number of days from inoculation to full substrate colonization; the line shows the corresponding average mycelial growth rates. BS – beechwood sawdust (control); WS – wheat straw (control). Explanations of abbreviations (A1–K12) are provided in the Supplementary Material (Table S1).

The results of the screening tests for all waste mixtures used are shown in Fig. 1. Waste mixtures K4-K6 did not completely colonize the substrate within 21 days of incubation. The control sample was a substrate containing only wheat straw or beech wood sawdust. For the control media, complete overgrowth of the substrate occurred on days 12 and 21, respectively. On most substrates, complete colonization was achieved within 21 days. Consequently, extending the experiment beyond this period was deemed unnecessary, as the mycelial growth phase had already concluded.

The fastest growth was observed for variant C1 (WS + BSG at a ratio of 70/30% d.m.), variant E1 (WS + WB at a ratio of 70/30% d.m.) and variant J2 (WS + WB + SBP at a ratio of 50/25/25% d.m.), respectively. Complete overgrowth of this substrate occurred on the 12th day of incubation already with AGR = 0.92 cm·day^−1^. Similar results were obtained for variant E2 (WS + WB in a ratio of 50/50% d.m.). However, this variant was excluded because its composition was already included in variant E1, but in different proportions, and our aim was to test differentiated substrate mixtures. Since the substrate used in E1 yielded comparable results while being more economical due to the lower proportion of wheat bran, we chose to proceed with the more cost-effective option. On the other substrates, the total colonization period was longer and ranged from 14 to 21 days, depending on the substrate used, with an average growth rate ranging from 0.44 to 0.79 cm·day^−1^. Therefore, further studies conducted on a larger scale (in polyethylene plastic bags) were continued only for the C1, E1, and J2 substrates.

**Fig. 2:**
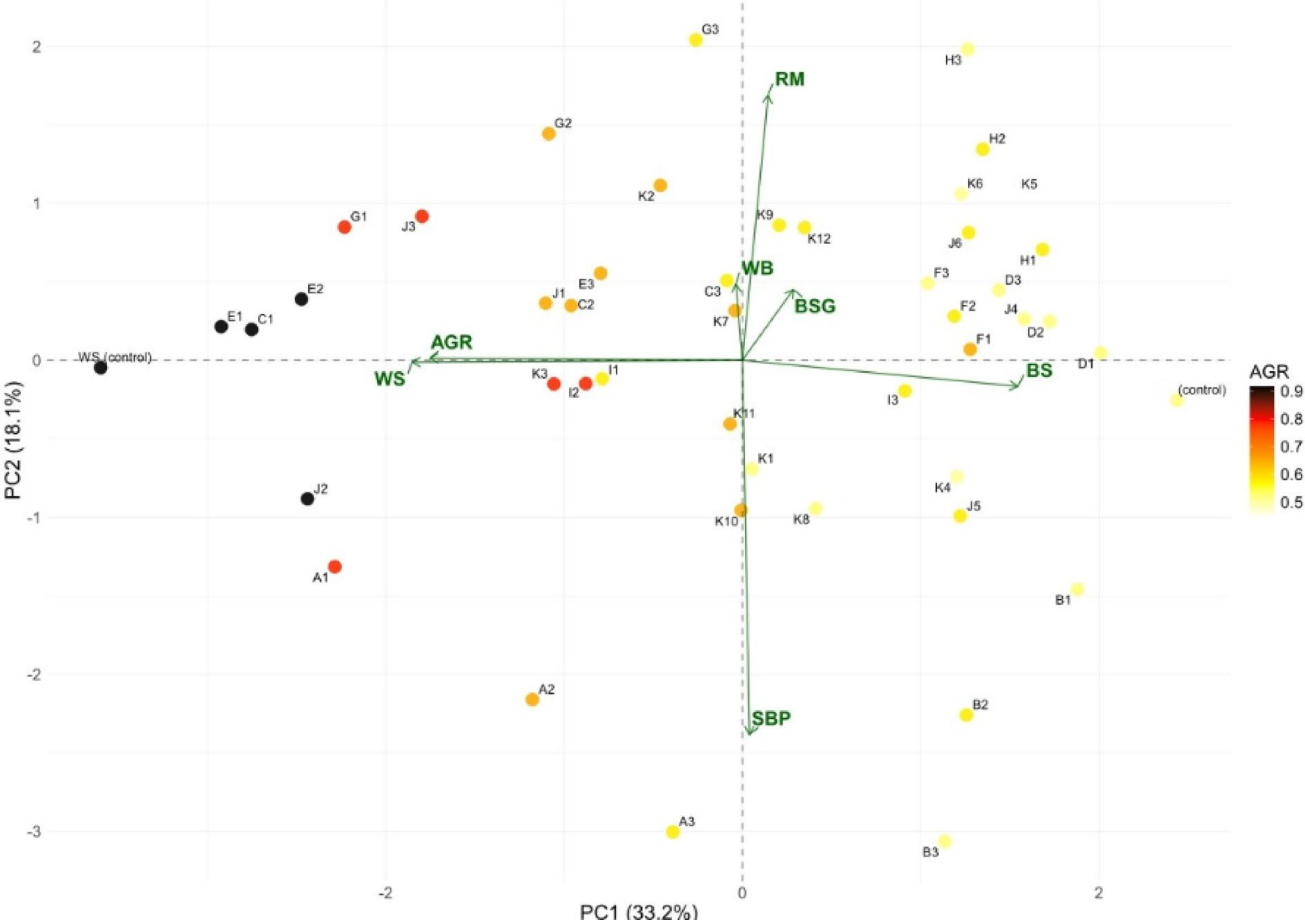
Principal component analysis (PCA) biplot showing the relationship between substrate types and average growth rate (AGR) of oyster mushroom (*Pleurotus ostreatus*). The analysis includes 11 substrate groups (A-K) with their respective subgroups: A1-A3, B1-B3, C1-C3, D1-D3, E1-E3, F1-F3, G1-G3, H1-H3, I1-I3, J1-J6, and K1-K12. (Table S1, supplementary materials). Points represent individual substrate compositions colored according to their AGR values (scale 0.5–0.9). Green arrows indicate environmental variables: AGR (average growth rate), RM (rapeseed meal), WB (wheat bran), BSG (brewer’s spent grain), WS (wheat straw), BS (beechwood sawdust), and SBP (sugar beet pulp). Control treatments (WS control and BS control) are marked in black. The proximity of substrate points to the AGR vector indicates their correlation with growth performance.

The PCA biplot (Fig. 2) explains 51.3% of the total variance (PC1: 33.2%, PC2: 18.1%) and reveals distinct clustering patterns of substrate compositions based on their correlation with average growth rate (AGR). Substrates positioned in the upper right quadrant, particularly groups H and D, demonstrate the strongest positive correlation with AGR, indicating optimal growth performance. The vector analysis shows that wheat bran (WB) and rapeseed meal (RM) exhibit strong positive correlations with growth rate, while beechwood sawdust (BS) shows a negative correlation, confirming that high lignin content impedes mycelial development. The 70/30% substrate compositions (70% wheat straw + 30% BSG or WB) that were selected for further investigation based on preliminary screening results are positioned favorably relative to the AGR vector, validating their superior colonization rates. These mixed substrates significantly outperformed single-component controls, with pure wheat straw (WS control) showing moderate performance near the origin and pure beechwood sawdust (BS control) demonstrating the poorest correlation with growth parameters.

**Fig. 3:**
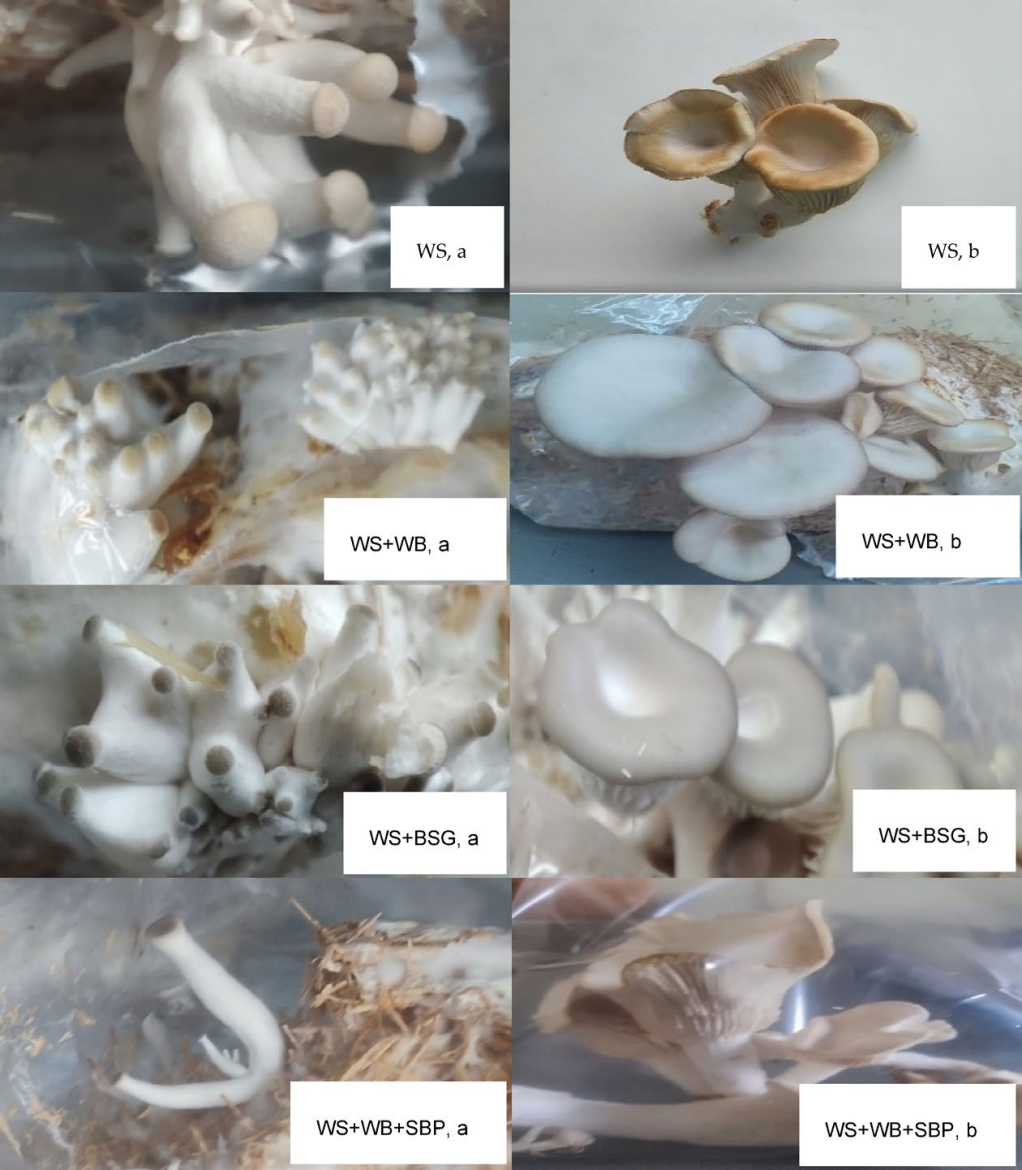
The growth of *P. ostreatus* on different waste mixtures (WS, wheat straw; WB, wheat bran; BSG, brewery spent grains; SBP, sugar beet pulp, a- formation of primordia; b- 1 st flush of fruiting bodies).

The results obtained from the study of the effect of different substrate compositions mixtures on morphological parameters and characteristics of oyster mushroom fruiting bodies are shown in Table 2. The appearance of fruiting bodies, both at the stage of primordia formation and during the first harvest, on different waste mixtures, is shown in Fig. 3.

**Table 2:** Effect of different substrate formulas on morphological parameters and characteristics of the fruiting body of *Pleurotus ostreatus* grown in polyethylene plastic bags (WS, wheat straw; WB, wheat bran; BSG, brewery spent grains; SBP, sugar beet pulp; BE, biological efficiency; ± means standard deviation for *N* = 3. Values for single element designated with the same letter are not significantly different (*P* < 0.05) according to tukey’s Kramer test for multiple comparisons).

Variables	Substrate formula			
	WS 100% (control)	WS + WB (70/30%)	WS + BSG (70/30%)	WS + WB + SBP (50/25/25%)
Total colonization period (day)	24.50 ± 0.71c	21.00 ± 0.00b	16.00 ± 0.71a	21.50 ± 1.41b
Formation of primordia (day)	32.00 ± 1.41c	27.50 ± 0.71b	20.50 ± 0.71a	28.00 ± 1.41b
First harvest (day)	35.50 ± 3.54b	39.00 ± 1.41c	27.50 ± 0.71a, c	30.00 ± 1.41b, c
Cap diameter (cm)	4.10 ± 1.14a	3.10 ± 0.79a	4.40 ± 0.74a	3.00 ± 1.14a
Stipe length (cm)	2.40 ± 0.89a	3.90 ± 0.85 a, b	5.10 ± 1.11b	3.90 ± 1.51a
No. of effective fruiting bodies	5.00 ± 1.41a	15.50 ± 7.78a	12.50 ± 3.54a	2.00 ± 1.41a
Mushroom weight (g·bag^− 1^)	39.00 ± 15.41a	40.60 ± 11.81a	51.70 ± 9.76a	20.0 ± 8.7a
Biological efficiency BE (%)	41.90 ± 1.25a	45.30 ± 1.41a	54.33 ± 1.53b	13.80 ± 1.25c

The fastest growth of fungi in bags was obtained on WS + BSG substrate (70/30% relative to substrate). Complete overgrowth of the medium occurred as early as day 16.00 ± 0.71 of incubation, which was faster by 8.5 days compared to the control sample. The first primordia appeared at 20.50 ± 0.71 days of incubation approximately, and the minimum duration required for first harvest was 27.50 ± 0.71 days (Table 3). Compared to the control sample (WS), where the first primordia were observed at day 32.00 ± 1.41 and the first harvest at 35.50 ± 3.54 days of incubation, the results for the above medium were promising. In the case of WS + WB (70/30%), and WS + WB + SBP (50/25/25%), complete overgrowth of the substrate occurred on day 21.00 ± 0.00 and 21.50 ± 1.41 of incubation, and the first primordia could be seen on day 27.50 ± 0.71 and 28.00 ± 1.41, respectively. The average time for harvesting the first primordia, however, differed, depending on the type of culture medium used. Differences were observed in the quality and quantity of the fruiting bodies produced. The fruiting bodies on the WS substrate grew singly, and their weight after weighing averaged 39.00 ± 15.41 g·bag^− 1^, the cap diameter averaged 4.10 ± 1.14 cm, and the stipe length was about 2.40 ± 0.89 cm. In the case of WS + WB substrate, in the first flux, fruiting bodies were harvested with a total weight of 40.60 ± 11.81 g·bag^− 1^ on average, cap size on average 3.10 ± 0.79 cm and with a stipe length of 3.90 ± 0.85 cm on average. For the WS + BSG substrate, after the 1 st flush, it was possible to obtain the highest yield equal to 51.70 ± 9.76 g·bag^− 1^ on average. The fruiting bodies on this substrate were characterized by excellent vigor, the caps were not curled, the size of about 4.40 ± 0.74 cm (Fig. 1). No significant differences in cap diameter were found between the experimental variants. On the WS + WB + SBP substrate, growth was the least intensive. The yield after the 1 st flush did not exceed 20.0 ± 8.7 g·bag^− 1^, and the fruiting bodies were devoid of vigor and curled inward (Fig. 3).

The biological efficiency (BE) of the fruiting process, defined as, the ratio of fungal yield to substrate weight, for the three selected waste mixtures, is also shown in Table 2. Statistically significant differences in BE values were observed between the various types of applied waste substrates. The WS + BSG substrate demonstrated the highest biological efficiency, with a BE rate of 54.33 ± 1.53%, which was much higher than the control substrate (41.90 ± 1.25%). The waste from brewing is a lignocellulosic material, high in protein and fiber, so it is reasonable to consider it a suitable substrate for fungal growth. The WS + WB substrate had a biological efficiency of 45.30 ± 1.41%. The results for the WS + WB + SBP substrate were not as promising, this substrate had an efficiency of only 13.80 ± 1.25%. Taking into account that the WS + BSG and WS + WB substrates contain 70% wheat straw, while the WS + WB + SBP substrate contains only 50%, it can be concluded that the lignocellulosic composition of the substrate plays an important role in fungal productivity. For instance, wheat straw alone contains 35.73% cellulose and 6.65% lignin, providing a favorable cellulose-to-lignin ratio for enzymatic degradation^31^. A well-balanced substrate with sufficient cellulose and moderate lignin enhances mycelial growth and fruiting, underscoring the importance of selecting and formulating substrates based on their chemical composition.

A similar study was presented by Keneni and Beka (2024) in their work, using various combinations of pea straw with wheat bran and cellulosic material in the form of used paper^32^. The fastest growth was obtained for a substrate containing pea straw, paper and wheat bran at a ratio of 40/40/20% and for a substrate containing paper, straw and wheat bran at a ratio of 35/35/30%. Complete overgrowth of the substrate occurred within 12 days, and primordia formation occurred as early as day 17 of incubation, a time that was 13 days shorter compared to the control substrate^32^. Hendrika et al. (2021) using different combinations of culture media containing sawdust, rice bran, grass straw, rice husk, among others, proved that media containing 100% sawdust or 100% grass straw can be an alternative base material for growth media. However, on these media, complete colonization of the substrate occurred not until the 21 st day^20^. Petsov et al. (2021) proved that the culture medium containing waste from the brewery industry, gave the fastest growth of *P. ostreatus*. After 7 days of incubation, the mycelium occupied 9% more Petri dish surface area than the traditionally used PDA (Potato Dextrose Agar) medium^33^. Also, Fufa et al. (2021), in their work, showed a relationship between the type of substrate and the yield obtained. Studying the growth capacity of *P. ostreatus*, on substrates containing corn cobs, palm millet straw and bamboo waste, they showed that the best biological yield was the substrate with the addition of palm millet straw. They obtained an efficiency of 50%. The substrate, which was a mixture of corn cobs with bamboo waste added, yields was 20% only^34^. Maheswaeri et al. (2020) cultivated *P. ostreatus* on various waste compositions, including: corn casing/corn cob/coconut pith, paddy straw/ragi straw and sugarcane bagasse supplemented with 10% wheat bran. The fastest mycelial growth was obtained for the substrate that was a mixture of the two types of straw on the 28th day of incubation already. The BE rate was also the highest for this substrate and was as high as 92.08%, while the yield for the substrate containing the cover and corn cobs was about 72%^35^. In a study by Wang et al. (2001) on the growth capacity of *P. ostreatus* on waste from the brewing industry, in turn, it was proven that few fruiting bodies were formed on grain milling alone, but a much higher biological yield (19.1%) was obtained when wheat bran (45%) was added^36^. All of the above work confirms that the yield capacity, as well as the quality of the fruiting bodies produced, depends largely on the composition of the substrate used to cultivate oyster mushrooms^37^. In this work, the most effective substrates were the cereal mixture with the addition of brewery spent grains (WS + BSG) and wheat bran in proportions of 70/30% (WS + WB), while the mixture of 50% wheat straw, 25% wheat bran, and 25% sugar beet pulp (WS + WB + SBP) yielded slightly weaker results. However, the BE rate was at a satisfactory level only for the first two mixtures, equaling 54.33 ± 1.53% and 45.30 ± 1.41% for the WS + BSG and WS + WB substrates, respectively (Table 2). Wheat straw with a high proportion of cellulose proved to be the key ingredient. When added at 70% in substrate, it supported effective *P. ostreatus* colonization. It also provides suitable physical structure and moisture conditions that facilitate mycelial development. When combined with nutrient-rich additives such as spent grain or wheat bran, it forms a balanced substrate that promotes *Pleurotus* growth.

### Elemental content of *P. ostreatus* fruiting bodies and spent mushroom substrate

Dry mass, organic dry mass, ash and C and N content was studied in both *P. ostreatus* culture media and fruiting bodies (Table 3). The high C and N content was in WS + BSG medium. The content in carbon was about 50.20 ± 1.83%, while 2.00 ± 0.16% was nitrogen. Thus, the ratio of carbon to nitrogen, i.e., C/N, was about 24.73 ± 2.45, while in the sample before inoculation, the ratio was only 18.35 ± 2.19. For the WS + WB + SBP substrate, similar results were obtained. The C content was relatively high at 45.20 ± 1.40%, and the N content was in the low 1.70 ± 0.14% range. The C/N ratio in this case was 26.90 ± 7.52. The WS + WB substrate after fungal cultivation also showed differences compared to the control substrate. However, the carbon content was slightly lower than in the case of WS + BSG, at around 46.70 ± 2.32%, while the nitrogen content was only 1.10 ± 0.07%, about half the value observed for WS + BSG and comparable to WS + WB + SBP. As a result, the C/N ratio doubled (41.98 ± 3.37) and was the highest C/N value obtained in this study. However, it should be noted that this substrate, which contained wheat bran, experienced a nitrogen decrease comparable to that observed in other experiments (from 1.70 ± 0.81% to 1.10 ± 0.07%). In the case of fruiting bodies obtained after growth of *P. ostreatus* on the 3 selected substrates, different results were obtained. The carbon content in the fruiting bodies obtained from the WS + BSG substrate reached 53.90 ± 1.35%, followed by 49.80 ± 2.99% in those from the WS + WB substrate, and 46.20 ± 2.43% in those from the WS + WB + SBP substrate. No statistically significant differences were observed for fruiting bodies in the test for N content, which reached a value of about 2% for all samples tested. The C/N ratio for the fruiting bodies harvested from substrate supplemented with brewery spent grains (WS + BSG) was about 22.33 ± 2.05, with wheat bran (WS + WB) about 30.37 ± 6.39, and for the substrate with both wheat bran and sugar beet pulp (WS + WB + SBP) was about 26.65 ± 2.27. The study proved that the C/N ratio of the initial mixtures used for *P. ostreatus* cultivation, at the level of 18.35 ± 2.19, favorably affects the mycelial growth and promotes fruiting bodies production. A study by Sözbir et al. (2015) on the cultivation of *Pleurotus ostreatus* demonstrated that substrates with C/N ratios of 19.00 and 22.00 resulted in the highest biological efficiency and yield, suggesting that C/N values close to the lower end of this range (~ 19) are optimal for mycelial growth and fruiting body production^38^. The 1.7 ± 0.14–2.3 ± 0.72% N content has a beneficial effect on the quality and quantity of *P. ostreatus* yields in these mixtures. The higher carbon content of primary mixtures, on the other hand, results in lower biological efficiency. Additionally to highlight the differences in the C/N ratio across various substrates and growth stages (substrate before cultivation, after cultivation, and fruiting bodies), a bar chart was generated and included in the supplementary materials (Figure S3 – supplementary materials).

**Table 3:** Physical and chemical properties of the fruiting bodies and *Pleurotus* spent mushroom substrate. (WS, wheat straw; WB, wheat bran; BSG, brewery spent grains; SBP, sugar beet pulp, SMS- spent mushroom substrate, FB- fruiting bodies; ± means standard deviation for *N* = 3. Values for single element designated with the same letter are not significantly different (*P* < 0.05) according to tukey’s Kramer test for multiple comparisons).

Substrate formula	Dry matter [%]	Ash content [%]	Organic dry matter [%]	Carbon content [%]	Nitrogen content [%]	Carbon/Nitrogen Ratio
WS + BSG (control)	52.82 ± 0.28a	0.03 ± 0.02a	53.30 ± 0.55a	42.40 ± 0.75a	2.30 ± 0.72a	18.35 ± 2.19a
WS + WB (control)	34.34 ± 0.38b	0.01 ± 0.01a	35.55 ± 0.74b	49.80 ± 2.57b	1.70 ± 0.81a	29.62 ± 3.63b
WS + WB + SBP (control) (control)	26.30 ± 0.16c	0.01 ± 0.01a	26.36 ± 0.51c	51.30 ± 1.25b	2.60 ± 0.24a	19.44 ± 1.35a
WS + BSG SMS	34.04 ± 0.05b	4.16 ± 0.08b	29.88 ± 0.12c	50.20 ± 1.83b	2.00 ± 0.16a	24.73 ± 2.45c
WS + WB SMS	33.20 ± 1.88b	4.82 ± 0.63c	28.38 ± 1.33c	46.70 ± 2.32a	1.10 ± 0.07b	41.98 ± 3.37d
WS + WB + SBP SMS	35.14 ± 1.64b	1.64 ± 0.22d	33.50 ± 1.55b	45.20 ± 1.40a	1.70 ± 0.14a	26.90 ± 7.52c
WS + BSG FB	20.59 ± 0.07d	1.28 ± 0.11d	19.52 ± 0.30b	53.90 ± 1.35b	2.40 ± 0.27a	22.33 ± 2.05c
WS + WB FB	21.27 ± 0.26d	0.03 ± 0.02a	21.26 ± 0.20b	49.80 ± 2.99b	1.60 ± 0.17a	30.37 ± 6.39b
WS + WB + SBP FB	29.18 ± 0.259e	0.05 ± 0.03a	29.72 ± 0.39c	46.20 ± 2.43a	1.70 ± 0.10a	26.65 ± 2.27c

**Fig. 4:**
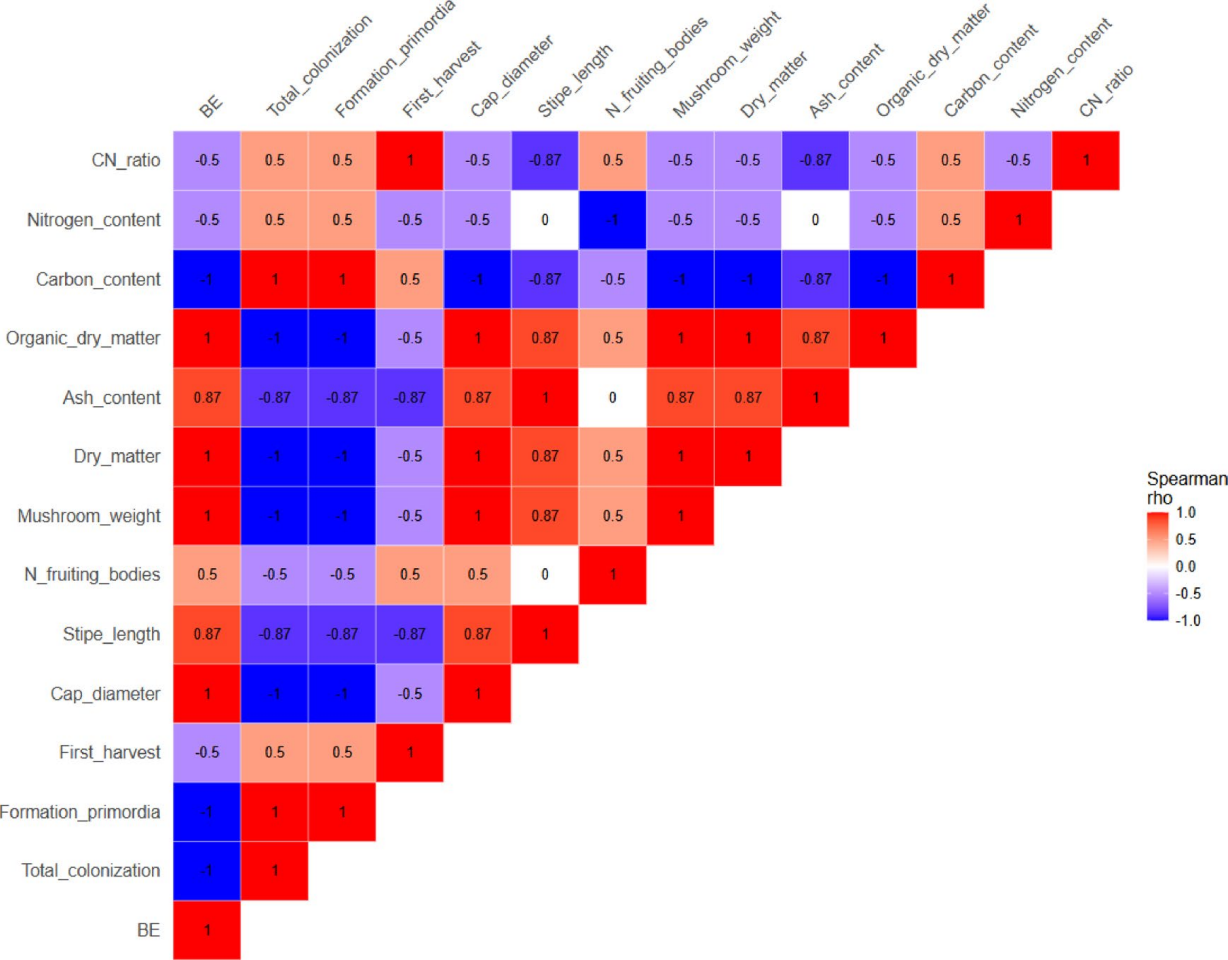
Spearman’s rank correlation matrix showing relationships between substrate physicochemical properties and *P. ostreatus* morphological and yield parameters. Colour scale indicates the direction and strength of correlations (ρ), from strong negative (blue) to strong positive (red).

Statistical analysis of the data presented in Tables 2 and 3 revealed a strong negative correlation between the carbon-to-nitrogen (C/N) ratio and biological efficiency (BE), mushroom weight, stipe length, and cap diameter (Fig. 4). This suggests that lower C/N ratios are more favorable for successful cultivation. Similarly, high carbon content was negatively correlated with morphological traits such as cap diameter, stipe length, BE, and both the number and weight of fruiting bodies, while a positive correlation was observed with total colonization time and the time to primordia formation. Finally, nitrogen content exhibited a moderate positive correlation with total colonization time, primordia formation, and selected morphological parameters. Several other variables showed weak or negligible correlations, indicating low variability among the tested substrates. Additional information about connection of the variables has been added in supplementary material (Figure S4).

Numerous studies have confirmed the significant impact of C/N ratio in the cultivation substrate on mycelial growth and fruiting of *Pleurotus* species, which is consistent with the findings of the present study. Philippoussis et al. (2001) emphasized that an appropriate C/N ratio is crucial for the rate of lignocellulose degradation and mushroom yield^31^. In our study, the highest yield was obtained using the WS + BSG substrate (Table 2, Biological Efficiency BE: 54.33 ± 1.53%), characterized by a higher carbon content and a relatively low C/N ratio (24.73 ± 2.45). In contrast, the WS + WB substrate, with lower nitrogen content and a higher C/N ratio (41.98 ± 3.37), produced a lower yield (Table 2, BE: 45.30 ± 1.41%). These results are consistent with those reported by Zakil et al. (2022), who highlighted the importance of the availability of carbohydrates in cellulose-based substrates for effective mycelial development^39^. However, the findings of Zhou et al. (2022) differ from ours. They reported that substrates with higher C/N ratios, such as lumber dust (C/*N* = 53:0.1), yielded higher productivity and biological efficiency compared to substrates with lower C/N ratios (e.g., cotton waste, C/*N* = 39:1)^40^. Similarly to our study, Lee et al. (2021) also confirmed the significant role of nitrogen content in *P. ostreatus* cultivation. They observed that the highest yields were achieved at nitrogen concentrations of 2.0–2.2% and C/N ratios of 20–22.5^41^. In contrast, our results suggest that lower nitrogen levels, ranging from 1.1% to 1.7%, were favorable for fruiting body formation on the tested substrates. Colla et al. (2023) demonstrated that higher nitrogen content increases laccase activity, which supports mycelial development, whereas reduced enzyme activity may initiate fruiting body formation^42^. Furthermore, Cueva et al. (2017) found a significant effect of nitrogen content (0.5–1.4%) on both the productivity and protein content of *P. ostreatus* fruiting bodies. The most favorable results were achieved with a substrate containing 1% nitrogen and a C/N ratio of 47.99, further confirming the importance of optimizing the balance between carbon and nitrogen for efficient *P. ostreatus* cultivation^43^.

The results of the studies of elemental content of *Pleurotus ostreatus* fruiting bodies and SMS are shown in Table 4. Studies showed the presence of valuable macro- and micronutrients, however statistically significant differences were observed depending on the type of substrate used.

**Table 4:** Elements content in fruiting bodies and *Pleurotus* spent mushroom substrate [mg·kg^−1^ d. m. substrate] **(**WS, wheat straw; WB, wheat bran; BSG, brewery spent grains; SBP, sugar beet pulp, SMS- spent mushroom substrate, FB- fruiting bodies; ± means standard deviation for *N* = 3. Values for single element designated with the same letter are not significantly different (*P* < 0.05) according to tukey’s Kramer test for multiple comparisons).

Substrate formula*	Ca	Cu	Fe	K	Mg	Mn	*P*	S	Zn
WS + BSG (control)	3674.94± 196.06a	14.92 ± 1.37a	119.56 ± 8.60a	4795.46 ± 136.03a	943.90 ± 25.50a	22.26 ± 0.80a	2259.09 ± 86.47a	553.78 ± 17.10a	65.89 ± 2.25a
WS + WB (control)	2738.42 ± 103.55 b	9.59 ± 1.07a	111.58 ± 15.26a	6047.33 ± 119.94a	1006.42 ± 65.23a	33.74 ± 1.86b	2441.12 ± 68.44a	366.40 ± 32.85a	58.85 ± 1.78a
WS + WB + SBP (control)	3243.45 ± 165.66a	7.82 ± 0.83a	97.30 ± 7.52a	5624.80 ± 141.47a	958.57 ± 89.06a	31.96 ± 2.33b	2532.34 ± 143.86a	533.98 ± 57.66a	32.78 ± 2.41b
WS + BSG SMS	6114.86 ± 136.46 c	26.67 ± 1.05b	112.07 ± 6.51a	1532.15 ± 22.25b	857.07 ± 33.09a	26.21 ± 1.40c	670.28 ± 15.53b	1652.77 ± 100.13b	58.23 ± 1.79a
WS + WB SMS	28907.68 ± 3689.24d	11.33 ± 1.18a	285.28 ± 11.19b	3447.16 ± 126.18c	1740.69 ± 83.64b	80.12 ± 0.76d	2255.48 ± 105.17a	6135.65 ± 701.08c	85.12 ± 3.80a
WS + WB + SBP SMS	12599.20 ± 288.61e	35.65 ± 3.58c	299.12 ± 22.03b	4576.01 ± 9.56a	1340.63 ± 88.37c	55.59 ± 0.48e	2932.90 ± 72.16a	2614.09 ± 193.24d	126.56 ± 3.96c
WS + BSG FB	993.09 ± 22.81f	17.73 ± 0.86 a. b	93.23 ± 3.86a	13953.74 ± 74.15 d	1137.72 ± 13. 81a.d	8.92 ± 0.90f	12708.46 ± 846.80c	1289.26 ± 136.85b	80.57 ± 7.38a
WS + WB FB	1112.62 ± 110.58f	24.09 ± 3.05b	145.82 ± 4.19c	16378.58 ± 747.74e	1573.81 ± 56.65b	12.51 ± 1.59f	18276.88 ± 242.92d	1528.28 ± 98.08b	182.02 ± 20.41d
WS + WB + SBP FB	3016.16 ± 257.13a	63.69 ± 1.74d	174.40 ± 5.73d	17255.60 ± 1111.15e	1742.29 ± 65.92b	11.58 ± 0.72f	11929.92 ± 422.71c	1371.28 ± 79.58b	200.29 ± 5.08d

The WS + BSG substrate after cultivation (SMS) contained large amounts of Ca, K, Mg and S. The highest value of the elements that were tested for this mixture was recorded for calcium, which was 6114.86 ± 136.46 mg·kg^−1^ d. m. substrate. Compared to the control substrate, this amount was almost twice as high. The content of sulfur increased 3-fold after growing *P. ostreatus* on a substrate supplemented with spent brewery grains. The substrate containing wheat bran (WS + WB) also showed an increase in the content of valuable nutrients after the culture stage. The amount of calcium increased almost 10-fold, with respect to the control medium without mycelium, and was approximately 28907.68 ± 3689.24 mg·kg^−1^ d. m. substrate. The amount of Mg increased by about 750 mg, and S increased almost 16-fold. These values for the substrate containing straw supplemented with wheat bran and sugar beet pulp (WS + WB + SBP) also differed significantly with the control substrate without mycelium. The average calcium content was 12599.20 ± 288.61 mg kg d.m.^−1^ substrate, and the Mg with S were 1340.63 ± 88.37 and 2614.09 ± 193.24 mg·kg^−1^ d.m. substrate, respectively.

The situation is different in the case of the elements K and P. Based on the analysis of K and P content in three types of substrates before and after mushroom cultivation and in their fruiting bodies, it can be stated that both elements were intensively taken up from the substrate and accumulated in the fruiting bodies, although to a different extent depending on the type of substrate. Potassium decreased significantly in all substrates, e.g. in substrate WS + BSG it dropped from 4795.46 ± 136.03 to 1532.15 ± 22.25 mg·kg^−1^ d. m. substrate, and in substrate WS + WB from 6047.33 ± 119.94 to 3447.16 ± 126.18 mg·kg^−1^ d. m. substrate. In turn, a very high potassium content was found in the fruiting bodies, ranging from 13953.74 ± 74.15 to 17255.60 ± 1111.15 mg·kg^−1^ d. m. substrate. In the case of phosphorus, a significant loss was observed mainly in substrate WS + BSG, while in substrates WS + WB and WS + WB + SBP the levels remained relatively stable or even increased, which may indicate a varied bioavailability of this element. Nevertheless, phosphorus, as well as potassium, was intensively accumulated in the fruiting bodies, especially in substrate WS + WB (18276.88 ± 242.92 mg·kg^−1^ d. m. substrate). These results confirm that both potassium and phosphorus play an important role in the metabolism of fungi, and that the appropriate selection of the substrate can affect their content in the final yield. For the other components, the amounts of elements tested in fruiting bodies were lower or comparable to the *Pleurotus* spent mushroom substrate.

Numerous scientific studies show that the mineral composition of fruiting bodies and substrates after *P. ostreatus* cultivation varies depending on the type of waste materials used^44–46^. Chemical analysis of fruiting bodies made by Wang et al. (2001) showed that *P. ostreatus* grown on spent beer grain had a higher nutritional value than those grown on other described types of substrates^36^. In the work of Sales-Campos et al. (2009), it was proven that after *P. ostreatus* cultivation on substrates containing sawdust or sugarcane, the K content reached various values ranging from 18 to 42 g·kg^−1^. It was also observed that the protein and mineral content of the used substrate increased compared to the initial substrate^44^. The relationship between the composition of the culture medium and the chemical composition of the fruiting bodies produced was also demonstrated by Sopanrao et al. (2010) in their study. Maximum protein, fat, ash, P, K and Na contents were recorded when *P. ostreatus* was grown on soybean straw alone. In contrast, maximum Ca and Fe contents were found on the substrate, which was a mixture of soybean and rice straw. In the *P. ostreatus* culture medium containing only soybean straw, on the other hand, they observed a decrease in the content of cellulose, hemicellulose, crude fiber, carbohydrates, lignin and tannins, while the content of protein, ash and minerals increased significantly^45^. Mortada et al. (2020), on the other hand, proved that the content of phosphorus, calcium, magnesium, iron and copper increased significantly in the spent substrate after cultivation of *P. ostreatus*^46^. In summary, the mineral content of the fruiting bodies and substrates after cultivating *P. ostreatus* varies depending on the type of waste materials used. Additionally, this study demonstrated that *Pleurotus* spent mushroom substrate is rich in valuable elements, highlighting its agricultural potential.

### Substrate decomposition

 Enzymatic activity of *P. ostreatus* was measured in extracts obtained from the medium after culture on: straw supplemented with brewery spent grains, wheat bran, and straw supplemented with wheat bran and beet pulp. The control sample was the substrate extract after mycelial culture on straw alone. The result of *Pleurotus* spent mushroom substrate extracts enzyme activity is shown in Table S3 (supplementary materials).

**Fig. 5:**
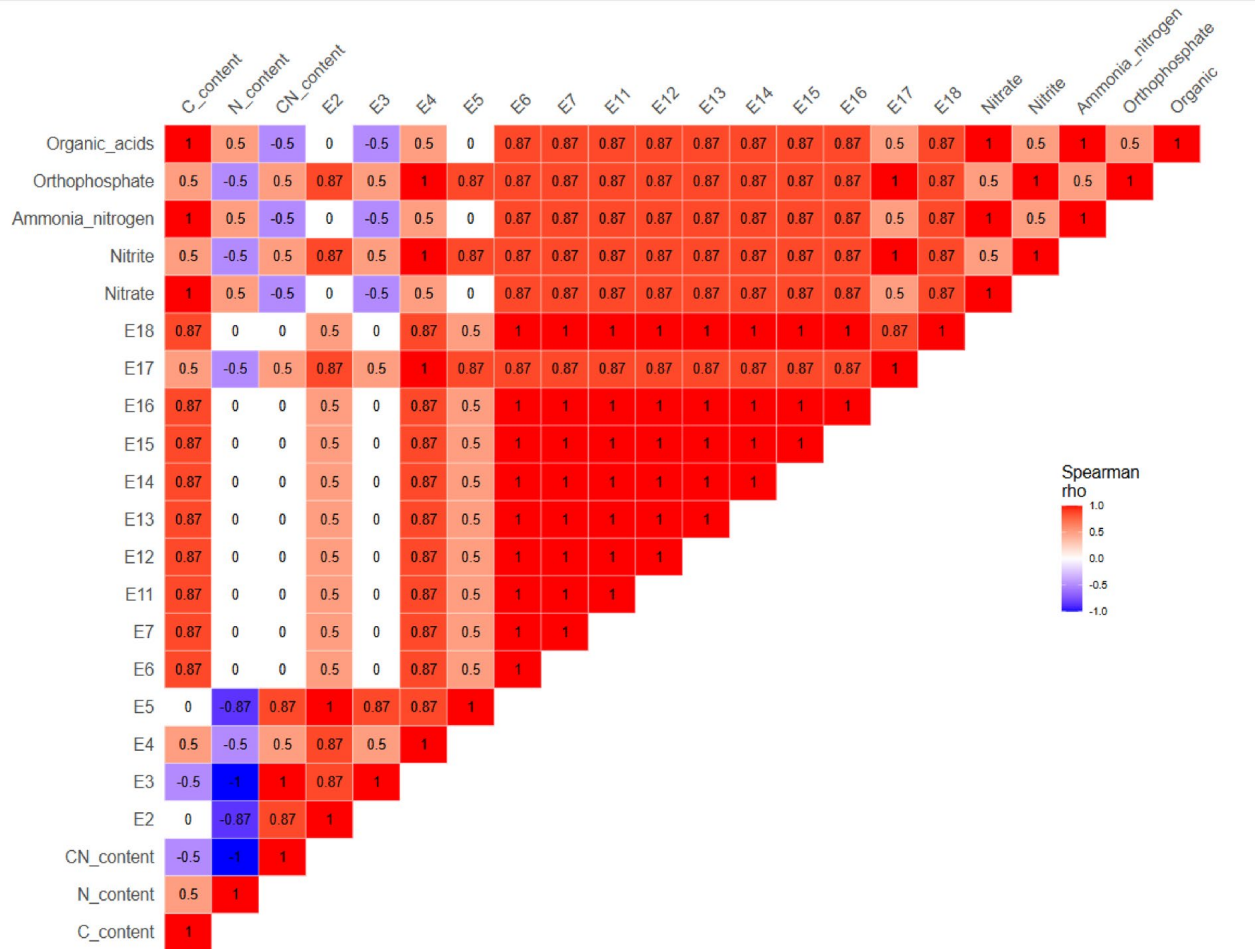
Spearman’s rank correlation matrix showing relationships between substrate physicochemical properties, substrate decomposition products level and *P. ostreatus* enzymatic activity. Colour scale indicates the direction and strength of correlations (ρ), from strong negative (blue) to strong positive (red). The explanations of the enzyme symbols E1-E20 are located in Table S3, supplementary materials.

High activities of leucine arylamidase (E6), acid phosphatase (E11), β-galactosidase (E14) and β-glucoronidase (E15) were found in *P. ostreatus* SMS containing straw and brewery spent grains extract (WS + BSG). However, there were no differences compared to the control medium. In contrast, the addition of brewery spent grains resulted in an increase in the activity of α -glucosidase (E16), valine arylamidase (E7), naphthyl-AS-BI phosphohydrolase (E12), α -galactosidase (E13), compared to the control medium. The activity of alkaline phosphatase (E2), esterase (E3), lipase (E5) decreased. An increase in the activity of valine arylamidase (E7), a slight increase in color intensity for naphthyl-AS-BI phosphohydrolase (E12) and β -galactosidase (E14) were observed in the medium with the addition of wheat bran (WS + WB). The activity of α-galactosidase (E13) increased from 1 to 3, and α -glucosidase (E16) increased from 0 to 3. For the medium supplemented with wheat bran and sugar beet pulp (WS + WB + SBP), no increase in the activity of either enzyme was observed. The extract, however, showed a slight activity of 1–2 for: esterase lipase- C8 (E4), leucine arylamidase (E6), acid phosphatase (E11), naphthyl-AS-BI phosphohydrolase (E12), β -galactosidase (E14), β-glucosidase (E17). At level 3, activity was observed for esterase (E3), and β-glucoronidase (E15). The best results were obtained for the medium supplemented with brewery spent grains. The activity of enzymes primarily responsible for the breakdown of sugars was found here. A relationship between the C, N content in SMS and the increase in enzymatic activity during growth was found. The higher C and N content in SMS, the more enzymes the fungus must produce to incorporate the elements into its structure. Statistical analysis of the data presented in Fig. 5 revealed a strong positive correlation between the carbon-to-nitrogen (C/N) ratio and the activity of enzymes E2, E3, and E5, while a moderate positive correlation was observed with enzymes E4 and E17. This suggests that higher C/N ratios may favor enzymatic activity in *Pleurotus ostreatus*. A particularly strong positive correlation (ρ = 0.87) was observed between carbon content and the activity of enzymes E6, E7, E11, E12, E13, E14, E15, and E16. In contrast, high nitrogen content was negatively correlated with the activity of enzymes E4 and E17. Additional information about connection of the variables has been added in supplementary material (Figure S5).

A similar study on the enzymatic activity of different species of Basidiomycota fungi was conducted by Homolka et al. (2010)^47^. Examining the medium after culturing the mycelium on perlite, they showed that *P. ostreatus* had the highest activity of the enzyme’s acid phosphatase, β -galactosidase, β -glucuronidase. However, the change in color intensity ranked 4 for valine arylamidase, α -galactosidase, β -glucosidase, and N-acetyl- β -glucosaminidase (Homolka et al., 2010). Eichlerová et al. (2015) proved that Basidiomycota showed higher activity of all enzymes, except alkaline phosphatase, α-glucosidase, N-acetylglucosaminidase, α-mannosidase and α-fucosidase, than Ascomycota^48^. The role of enzymatic activity in breaking down organic matter should be explicitly stated. Enzyme activities provide information on evidence of fungi converting complex substrates to simple ones as nutrients for them.

The study also assessed the content of substrate decomposition products, including orthophosphate, nitrate, nitrite, ammonium nitrogen and organic acids (Table 5).

**Table 5:** Determined parameters for substrate decomposition products (WS, wheat straw; WB, wheat bran; BSG, brewery spent grains; SBP, sugar beet pulp, SMS- spent mushroom substrate, ± means standard deviation for *N* = 3. Values for single element designated with the same letter are not significantly different (*P* < 0.05) according to tukey’s Kramer test for multiple comparisons).

Substrate formula	Nitrate [mg/L]	Nitrite [mg/L]	Ammonia nitrogen [mg/L]	Orthophosphate [mg/L]	Organic acids [mg/L]
WS + BSG	4.71 ± 0.12a	0.074 ± 0.01a	9.26 ± 2.46a	81.10 ± 1.27a	267.05 ± 4.95a
WS + WB	4.68 ± 0.04a	0.04 ± 0.00a	3.69 ± 0.01a	73.15 ± 1.34a	255.00 ± 36.77a
WS + WB + SBP	4.82 ± 0.16a	0.04 ± 0.03a	7.14 ± 1.06a	63.20 ± 10.47a	357.50 ± 34.65a
WS + BSG SMS	33.05 ± 0.18b	0.32 ± 0.09b	303.00 ± 81.27b	298.33 ± 20.55b	1150.33 ± 36.07b
WS + WB SMS	27.62 ± 0.57c	0.35 ± 0.14b	69.53 ± 8.98a	314.67 ± 11.02b	857.67 ± 107.56c
WS + WB + SBP SMS	8.62 ± 0.37d	0.06 ± 0.01a	50.17 ± 6.15a	184.33 ± 19.43c	350.33 ± 37.29a

The highest increase for all the compounds tested was recorded for the substrate supplemented with spent brewery grains (WS + BSG). The content of organic acids increased from the level of about 267.05 ± 4.95 mg·L^−1^ to about 1150.33 ± 36.07 mg·L^−1^. The content of orthophosphates in the substrate after *P. ostreatus* culture was also at a relatively high level and reached a value almost 4 times higher compared to the substrate before culture. As for nitrogenous compounds, there was the greatest increase in ammonium nitrogen from about 9.26 ± 2.46 mg·L^−1^ to 303.00 ± 81.27 mg·L^−1^. The content of nitrates and nitrites, after culture, increased about 5 times. Satisfactory results were also obtained for *Pleurotus* SMS with wheat bran (WS + WB) extract. An 8-fold increase in nitrate content, a 6-fold increase in nitrite content and an increase in ammonia nitrogen levels from 3.69 ± 0.01 to 69.53 ± 8.98 mg·L^−1^ were recorded. The content of orthophosphate, compared to the control substrate, increased from 73.15 ± 1.34 to 314.67 ± 11.02 mg·L^−1^, and the content of organic acids increased almost 4 times. The lowest results were obtained for a substrate that was a mixture of wheat straw, wheat bran and sugar beet pulp (WS + WB + SBP). The values of nitrate and nitrite, compared to the control substrate, increased only twice. Ammonium nitrogen increased from 7.14 ± 1.06 to 50.17 ± 6.15 mg·L^−1^, and orthophosphate content increased from 63.20 ± 10.47 to about 184.33 ± 19.43 mg·L^−1^. No statistically significant differences were observed between the content of organic acids in this medium before and after *P. ostreatus* culture. Figure 5 shows that carbon content was strongly positively correlated with nitrate, ammonia nitrogen and organic acids, whereas nitrogen content was negatively correlated with orthophosphates and nitrites, but moderately positively correlated with nitrate, ammonia nitrogen and organic acids. These opposing patterns suggest that carbon- and nitrogen-rich substrates promote distinct nutrient cycling processes. This indicates that *P. ostreatus* modulates its metabolic pathways in response to substrate composition, likely optimizing enzymatic activity to access specific nutrient forms. Additional information about connection of the variables has been added in supplementary material (Figure S6).

The study conducted is important for understanding how fungi use different types of substrates as sources of nitrogen and phosphorus for growth. This makes it possible to optimize the substrate components and improve the growth efficiency of the cultivated fungi. Nitrogen, one of the most important bioactive elements, is transported into the living cell in inorganic (nitrates, nitrites, ammonia) or organic (amino acids, urea, other nitrogen compounds) forms. The key role is played by ammonia transported into the cell or formed in the cell as a metabolite of nitrates, nitrites, urea, amino acids, etc. Three main enzymes are involved in ammonia assimilation: glutamate dehydrogenase (GDH) (EC 1.4.1.4) and two enzymes used at low ammonia concentrations, glutamine synthetase (GS) (EC 6.3.1.2) and glutamate synthase (GOGAT) (EC 1. 4.1.13)^49^. In the conducted studies, the highest total levels of degradation products after the growth of *Pleurotus ostreatus* were observed in the substrate with the addition of spent brewery grains, which indicates its high capacity for organic material degradation and effectiveness in utilizing the tested substrate.

## Conclusions

The study confirmed that oyster mushroom post-culture substrates are rich in valuable nutrients, highlighting their potential as bio stimulants in agriculture. Changing the composition of the culture medium affects both the quantity of fungal yields and the characteristics of the fruiting bodies, including their micro- and macronutrient content. Supplementation of standard substrates with spent brewery grains or wheat bran, improves fruiting and accelerates the growth rate of the fungus. The content of valuable elements for plants also increases in the substrate after cultivation. Among the valuable elements present in the SMS are nitrogen (N), phosphorus (P), potassium (K), calcium (Ca), and magnesium (Mg), all of which play essential roles in plant nutrition and development. Additionally, the favorable carbon-to-nitrogen (C/N) ratio observed in the substrate after cultivation supports both mycelial activity and optimal conditions for plant growth. These nutrient-rich properties make SMS a promising soil amendment that can enhance soil fertility and overall crop productivity. However, further investigation is needed into the use of the optimized substrate mixtures developed in this study, particularly when combined with other beneficial or commonly used additives such as gypsum. Additionally, expanding research on substrate optimization through the incorporation of a broader range of agro-industrial wastes could enhance the sustainability and efficiency of *Pleurotus ostreatus* cultivation.

## Supplementary Information

Below is the link to the electronic supplementary material.

Supplementary Material 1

## Data Availability

The datasets used and/or analysed during the current study are available from the corresponding author on reasonable request.
