# Supplementary material for: Agri-food wastes as substrates for oyster mushroom (Pleurotus ostreatus) cultivation and their agricultural potential
Source: Sci Rep. 2025 Nov 28;15:42617. doi: 10.1038/s41598-025-26843-y (PMC12663327; doi:10.1038/s41598-025-26843-y)

**Table S1.** Waste mixtures used for *P. ostreatus* culture - screening of the substrates (WS-wheat straw, BS- beech sawdust, RM- rapeseed meal, SBP- beet pomace, WB - wheat bran, BSG- brewery spent grain).

| **Type of waste composition** | **Mixtures and ratio (%)** | | | | | |
| --- | --- | --- | --- | --- | --- | --- |
|  | **wheat straw (WS)** | **beech wood sawdust (BS)** | **rapeseed meal (RM)** | **sugar beet pulp (SBP)** | **wheat bran (WB)** | **brewery spent grain (BSG)** |
| A1 | 70% | - | - | 30% | - | - |
| A2 | 50% | - | - | 50% | - | - |
| A3 | 30% | - | - | 70% | - | - |
| B1 | - | 70% | - | 30% | - | - |
| B2 | - | 50% | - | 50% | - | - |
| B3 | - | 30% | - | 70% | - | - |
| C1 | 70% | - | - | - | - | 30% |
| C2 | 50% | - | - | - | - | 50% |
| C3 | 30% | - | - | - | - | 70% |
| D1 | - | 70% | - | - | - | 30% |
| D2 | - | 50% | - | - | - | 50% |
| D3 | - | 30% | - | - | - | 70% |
| E1 | 70% | - | - | - | 30% | - |
| E2 | 50% | - | - | - | 50% | - |
| E3 | 30% | - | - | - | 70% | - |
| F1 | - | 70% | - | - | 30% | - |
| F2 | - | 50% | - | - | 50% | - |
| F3 | - | 30% | - | - | 70% | - |
| G1 | 70% | - | 30% | - | - | - |
| G2 | 50% | - | 50% | - | - | - |
| G3 | 30% | - | 70% | - | - | - |
| H1 | - | 70% | 30% | - | - | - |
| H2 | - | 50% | 50% | - | - | - |
| H3 | - | 30% | 70% | - | - | - |
| I1 | 70% | 30% | - | - | - | - |
| I2 | 50% | 50% | - | - | - | - |
| I3 | 30% | 70% | - | - | - | - |
| J1 | 50% | - | - | - | 25% | 25% |
| J2 | 50% | - | - | 25% | 25% | - |
| J3 | 50% | - | 25% | - | 25% | - |
| J4 | - | 50% | - | - | 25% | 25% |
| J5 | - | 50% | - | 25% | 25% | - |
| J6 | - | 50% | 25% | - | 25% | - |
| K1 | 25% | - | - | 25% | 25% | 25% |
| K2 | 25% | - | 25% | - | 25% | 25% |
| K3 | 25% | - | 25% | 25% | - | 25% |
| K4 | - | 25% | - | 25% | 25% | 25% |
| K5 | - | 25% | 25% | - | 25% | 25% |
| K6 | - | 25% | 25% | - | 25% | 25% |
| K7 | 25% | 25% | - | - | 25% | 25% |
| K8 | 25% | 25% | - | 25% | 25% | - |
| K9 | 25% | 25% | 25% | - | 25% | - |
| K10 | 25% | 25% |  | 25% | - | 25% |
| K11 | 25% | 25% | 25% | 25% | - | - |
| K12 | 25% | 25% | 25% | - | - | 25% |
| BS (control) | - | 100% | - | - | - | - |
| WS (control) | 100% | - | - | - | - | - |


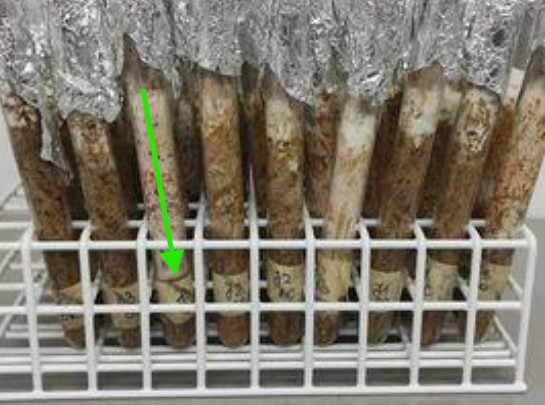


Figure S1. Fungal culture tests in test tubes. The green arrow shows the direction of fungal growth on the waste medium.

**Figure S2.** Growth curves of oyster mushroom (*Pleurotus ostreatus*) on different waste substrate mixtures. Mycelial growth is shown as a function of time for 11 substrate groups (A-K). Groups A-I contain three subgroups each (A1-A3, B1-B3, .... I1-I3). Group J contains six subgroups (J1-J6), and group K contains twelve subgroups (K1-K12). Each subgroup represents a different waste substrate (Table S1). The colour of the frame surrounding the growth curve equation and slope coefficient corresponds to the colour of the respective subgroups on the graph. Trend lines show the kinetics of mycelium biomass growth for individual substrate variants.

**Table S2.** Growth rate of oyster mushroom (*Pleurotus ostreatus*) on different waste substrate mixtures (calculated based on the linear model shown in Figure S2).

| Type of waste composition | Growth rate  [h^-1^] |
| --- | --- |
|  |  |
| A1 | 0.7908 |
| A2 | 0.6416 |
| A3 | 0.5434 |
| B1 | 0.5030 |
| B2 | 0.5136 |
| B3 | 0.4747 |
| C1 | 0.7688 |
| C2 | 0.5663 |
| C3 | 0.4566 |
| D1 | 0.5337 |
| D2 | 0.4346 |
| D3 | 0.4810 |
| E1 | 0.8433 |
| E2 | 0.7248 |
| E3 | 0.6031 |
| F1 | 0.5992 |
| F2 | 0.5494 |
| F3 | 0.4930 |
| G1 | 0.6990 |
| G2 | 0.5215 |
| G3 | 0.5216 |
| H1 | 0.5693 |
| H2 | 0.5821 |
| H3 | 0.5621 |
| I1 | 0.5284 |
| I2 | 0.7247 |
| I3 | 0.5782 |
| J1 | 0.5900 |
| J2 | 0.8529 |
| J3 | 0.7095 |
| J4 | 0.4212 |
| J5 | 0.5749 |
| J6 | 0.4982 |
| K1 | 0.5467 |
| K2 | 0.6424 |
| K3 | 0.6299 |
| K4 | 0.4737 |
| K5 | 0.3835 |
| K6 | 0.4539 |
| K7 | 0.6419 |
| K8 | 0.4685 |
| K9 | 0.5835 |
| K10 | 0.5949 |
| K11 | 0.6263 |
| K12 | 0.6299 |
| BS (control) | 0.5191 |
| WS (control) | 0.7436 |

**Figure S3.** C/N ratio in different *Pleurotus ostreatus* cultivation system: substrate before cultivation, substrate after cultivation, and fruiting bodies.


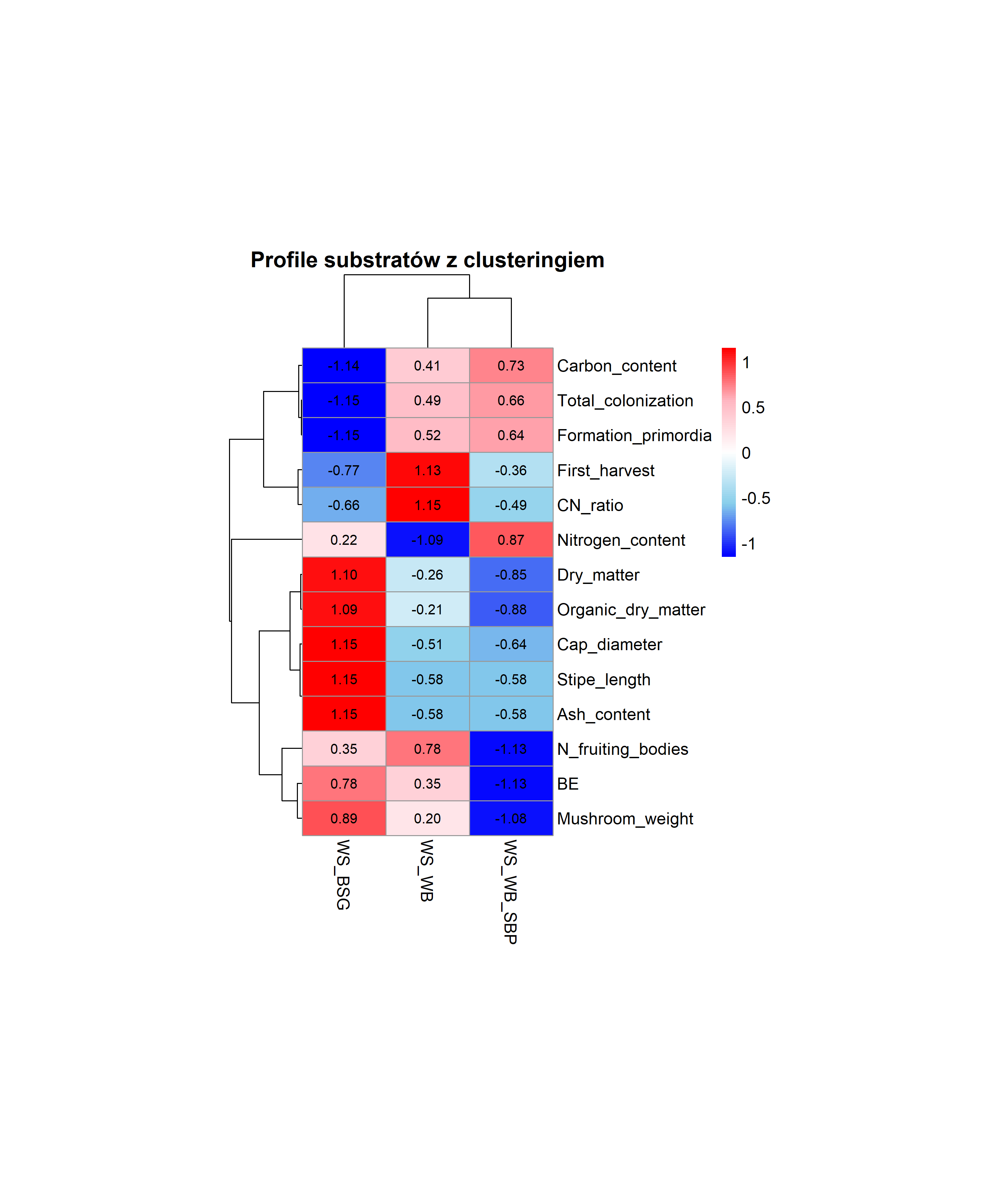


**Figure S4.** Substrate clustering heatmap showing the association between physicochemical properties of the substrates and morphological and yield parameters of the mushrooms (14 variables, n=3). The color scale ranges from -1 to 1 and reflects the relative magnitude of each variable within the dataset. The corresponding colors represent standardized values. where red indicates higher values and blue indicates lower values relative to the mean.


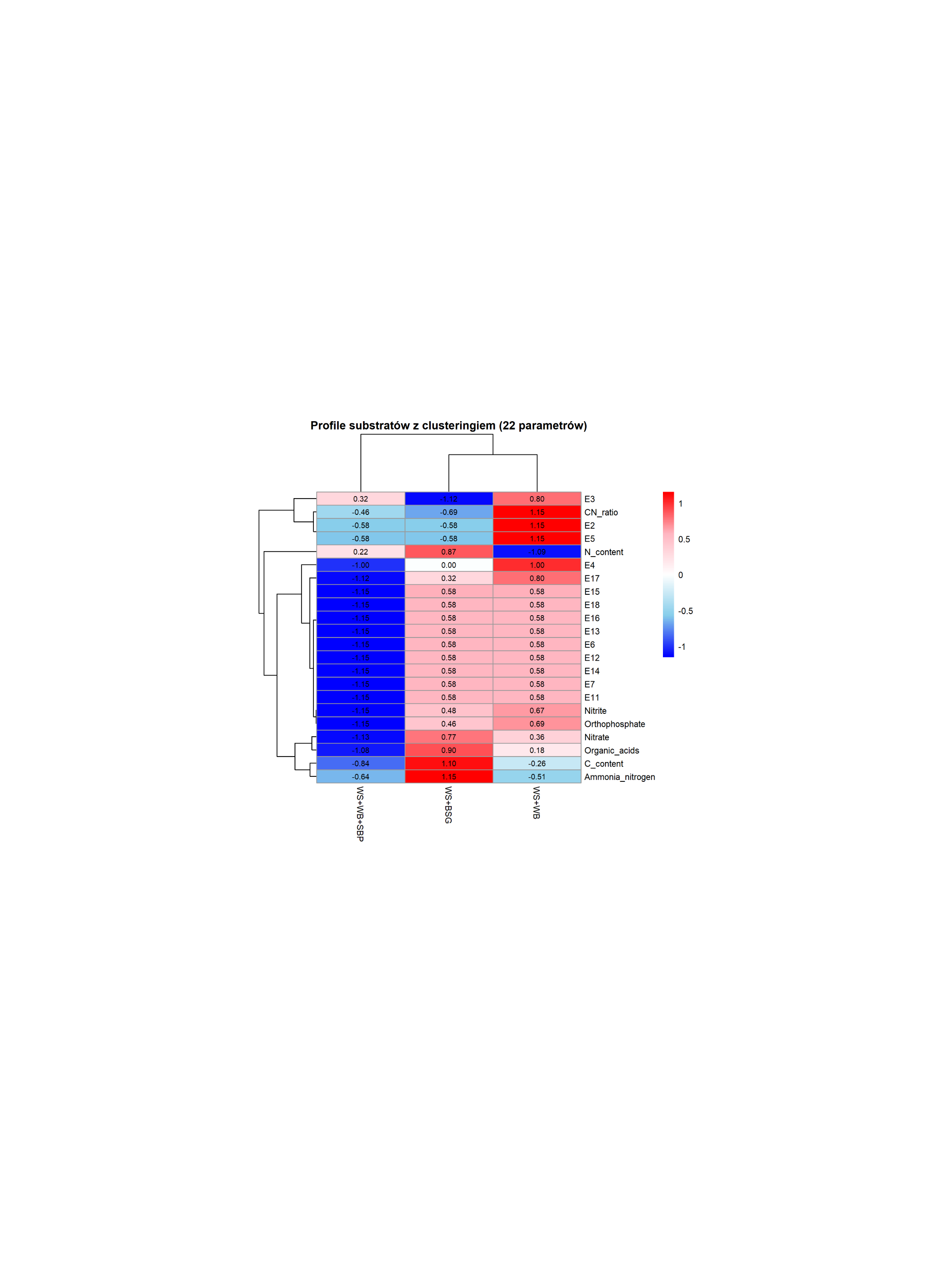


**Figure S5.** Substrate clustering heatmap showing the associations between enzyme activity and physicochemical properties of the substrates (22 variables, n=3). The colour scale ranges from -1 to 1 and reflects the relative magnitude of each variable within the dataset. The corresponding colours represent standardized values, where red indicates higher values and blue indicates lower values relative to the mean.

Table S3. Enzyme activity measured in *Pleurotus* spent mushroom substrate extracts (heat map). A score of 0 indicates no detectable enzyme activity, while a score of 5 represents the highest level of enzyme activity observed.

| **ENZYMES/symbols** | **WS+BSG** | **WS+WB** | **WS+WB+SBP** | **WS** |
| --- | --- | --- | --- | --- |
| Alkaline phosphatase/E2 | 0 | 4 | 0 | 4 |
| Esterase (C 4)/E3 | 0 | 4 | 3 |  |
| Esterase lipase (C 8)/E4 | 3 | 4 | 2 | 4 |
| Lipase (C 14)/E5 | 0 | 3 | 0 | 3 |
| Leucine arylamidase/E6 | 5 | 5 | 2 | 5 |
| Valine arylamidase/E7 | 4 | 4 | 0 | 1 |
| Cystine arylamidase/E8 | 0 | 0 | 0 | 0 |
| Trypsin/E9 | 0 | 0 | 0 | 0 |
| a-chymotrypsin/E10 | 0 | 0 | 0 | 0 |
| Acid phosphatase/E11 | 5 | 5 | 1 | 5 |
| Naphthol-AS-BI-phosphohydrolase/E12 | 4 | 4 | 1 | 3 |
| a-galactosidase/E13 | 3 | 3 | 0 | 1 |
| b –galactosidase/E14 | 5 | 5 | 1 | 4 |
| b -glucuronidase/E15 | 5 | 5 | 3 | 5 |
| a -glucosidase/E16 | 3 | 3 | 0 | 0 |
| b –glucosidase/E17 | 4 | 5 | 1 | 3 |
| N-acetyl- b -glucosaminidase/E18 | 3 | 3 | 0 | 3 |
| a –mannosidase/E19 | 0 | 0 | 0 | 0 |
| a -fucosidase/E20 | 0 | 0 | 0 | 0 |


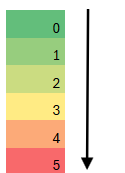

Supplement: Supplementary file 1 — Supplementary Material 1 [file 41598_2025_26843_MOESM1_ESM.docx]
